# Transfer RNA detection by small RNA deep sequencing and disease association with myelodysplastic syndromes

**DOI:** 10.1186/s12864-015-1929-y

**Published:** 2015-09-24

**Authors:** Yan Guo, Amma Bosompem, Sanjay Mohan, Begum Erdogan, Fei Ye, Kasey C. Vickers, Quanhu Sheng, Shilin Zhao, Chung-I Li, Pei-Fang Su, Madan Jagasia, Stephen A. Strickland, Elizabeth A. Griffiths, Annette S. Kim

**Affiliations:** Department of Cancer Biology, Vanderbilt University Medical Center, Nashville, TN USA; Department of Pathology, Immunology, and Microbiology, Vanderbilt University Medical Center, Nashville, TN USA; Department of Biostatistics, Vanderbilt University, Nashville, TN USA; Department of Medicine, Vanderbilt University Medical Center, Nashville, TN USA; Department of Applied Mathematics, National Chiayi University, Chiayi City, Taiwan; Department of Statistics, National Cheng Kung University, Tainan City, Taiwan; Roswell Park Cancer Institute, Elm & Carlton Sts, Buffalo, NY USA; Present address: Brigham and Women’s Hospital, 75 Francis Street, Boston, MA 02115 USA

**Keywords:** Transfer RNA (tRNA), microRNA (miRNA), Next generation (deep) sequencing (NGS), Predictive biomarkers, DNA methyltransferase inhibitor, Response to therapy, Myelodysplastic syndromes (MDS)

## Abstract

**Background:**

Although advances in sequencing technologies have popularized the use of microRNA (miRNA) sequencing (miRNA-seq) for the quantification of miRNA expression, questions remain concerning the optimal methodologies for analysis and utilization of the data. The construction of a miRNA sequencing library selects RNA by length rather than type. However, as we have previously described, miRNAs represent only a subset of the species obtained by size selection. Consequently, the libraries obtained for miRNA sequencing also contain a variety of additional species of small RNAs. This study looks at the prevalence of these other species obtained from bone marrow aspirate specimens and explores the predictive value of these small RNAs in the determination of response to therapy in myelodysplastic syndromes (MDS).

**Methods:**

Paired pre and post treatment bone marrow aspirate specimens were obtained from patients with MDS who were treated with either azacytidine or decitabine (24 pre-treatment specimens, 23 post-treatment specimens) with 22 additional non-MDS control specimens. Total RNA was extracted from these specimens and submitted for next generation sequencing after an additional size exclusion step to enrich for small RNAs. The species of small RNAs were enumerated, single nucleotide variants (SNVs) identified, and finally the differential expression of tRNA-derived species (tDRs) in the specimens correlated with diseasestatus and response to therapy.

**Results:**

Using miRNA sequencing data generated from bone marrow aspirate samples of patients with known MDS (*N* = 47) and controls (*N* = 23), we demonstrated that transfer RNA (tRNA) fragments (specifically tRNA halves, tRHs) are one of the most common species of small RNA isolated from size selection. Using tRNA expression values extracted from miRNA sequencing data, we identified six tRNA fragments that are differentially expressed between MDS and normal samples. Using the elastic net method, we identified four tRNAs-derived small RNAs (tDRs) that together can explain 67 % of the variation in treatment response for MDS patients. Similar analysis of specifically mitochondrial tDRs (mt-tDRs) identified 13 mt-tDRs which distinguished disease status in the samples and a single mt-tDR which predited response. Finally, 14 SNVs within the tDRs were found in at least 20 % of the MDS samples and were not observed in any of the control specimens.

**Discussion:**

This study highlights the prevalence of tDRs in RNA-seq studies focused on small RNAs. The potential etiologies of these species, both technical and biologic, are discussed as well as important challenges in the interpretation of tDR data.

**Conclusions:**

Our analysis results suggest that tRNA fragments can be accurately detected through miRNA sequencing data and that the expression of these species may be useful in the diagnosis of MDS and the prediction of response to therapy.

**Electronic supplementary material:**

The online version of this article (doi:10.1186/s12864-015-1929-y) contains supplementary material, which is available to authorized users.

## Background

RNA next generation sequencing (RNA-seq) technology has replaced microarrays as the primary platform for gene expression profiling [1–4]. This same technology has also been used to study the expression of microRNAs (miRNAs), although there are fewer direct comparisons between miRNA arrays and miRNA-seq [5–7]. During miRNA-seq library preparation, small RNAs are selected by electrophoresis on a size typically ranging from 20 to 50 nucleotides (nts). This range of size selection allows for the capture of many other species of small RNAs in addition to miRNAs [8–15].

One of the major byproducts of size selection for miRNAs is tRNA-derived small RNAs (tDRs) [13, 16]. This may be the result of either active cleavage or an artifact of the miRNA-seq library construction. Full length or parent tRNAs are adaptor molecules composed of RNA with a length typically ranging from 73 to 94 nts. They serve as the physical link that translates messenger RNA into protein. Cleavage of tRNAs by the RNAse III enzyme, angiogenin, may occur in a number of reactive conditions to produce tRNA-derived halves (tRHs) [17, 18]. Likewise, tRNAs can also be cleaved in a Dicer-dependent manner or as an *in vitro* phenomenon by incubation with MgCl_2_ or nuclease S1 [10, 19]. All of these processes result in cleavage within a hairpin loop. D loop cleavage results in a 19 nt tRNA-derived fragment (tRF), and cleavage of the anticodon loop before the anti-codon affords a 33 nt tRH [10, 19]. These 5’ tDR products, in particular the 33-base tRH, may be captured by miRNA-seq.

In addition, apparent tDRs and tRNAs with base errors found in RNA-seq datasets may arise from the extensive chemical modifications of parent tRNA nucleotides. While these modifications contribute physiologically to RNA stability, function, and structure [20], they may be either misincorporated during the requisite reverse transcription step [21], resulting in specific base errors [22], or result in blockage of reverse transcription, with production of a truncated cDNA product [23]. Therefore, sequencing datasets may contain many reads that appear to be tDRs, but in fact are actually derived from full-length parent tRNA templates.

The quantification of tRNAs and tDRs warrant study through high-throughput sequencing. Aberrations in tRNAs have been linked to a range of diseases, including neurological disorders [24–26], cancer [27–29], type II diabetes [30–32], and mitochondrial diseases [33–35]. In addition, tDRs have been identified in a number of cancer cell lines, and some tDRs may play defined biological roles, such as the role of tRF-1001 in proliferation [11]. Thus far, only one study has found a link between somatic mutations in mitochondrial tRNA (Leu (UUR), aka MT-TL1) and myelodysplastic syndromes (MDS) [36]. The potential functionality of tRNA in MDS remains largely unexplored.

As part of our efforts to study MDS, we have previously examined miRNA expression in MDS [37, 38]. MDS is a common disease of the elderly (median age 71–76) [39] characterized by ineffective maturation of hematopoietic cells. This ineffective maturation manifests clinically as low blood counts and morphologically as dysplasia. The MDS state has been associated with expression changes in miRNAs. Several entities have been discovered on 5q, which contribute to the disease phenotype in the 5q minus syndrome subtype of MDS [40]. miR-145 and miR-146 have recently been identified in the commonly deleted region, and their knockdown appears to be associated with the thrombocytosis and dysmegakaryopoiesis seen in 5q minus syndrome [41]. Researchers, including those in our group, have identified several other miRNAs whose expression is dysregulated with the diagnosis of other subtypes of MDS [37, 38, 42–45]. Published research on other types of small RNAs in MDS is limited.

MDS has a median survival of only 18–24 months [46], with death resulting from either cytopenic complications or transformation to acute myeloid leukemia (AML) [47]. Currently, there are only two FDA-approved drugs for the treatment of all types of MDS, both of which are DNA methyltransferase inhibitors (DNMTIs): 5-azacytidine, and decitabine. However, only 40–47 % of patients achieve the clinically meaningful responses of hematologic improvement or better with DNMTIs [48]. Although several recent studies have suggested the use of mutational analysis by DNA next generation sequencing (NGS, DNA-seq) [49], genetics [50], or the presence or absence of key pharmacodynamic and pharmacokinetic markers [51] to predict response, as yet, there are no clinical or laboratory parameters in routine clinical practice that accurately predict response to DNMTIs [52–54]. The methylation status of specific loci has been shown to be prognostically relevant in the treatment of a closely related disease, chronic myelomonocytic leukemia, by decitabine [55].

In this study, we examined the expression of tRNAs in paired pre- and post-treatment samples from patients with the diagnosis of MDS on receiving treatment with DNMTIs. In miRNA sequencing data, the abundance of tRNA fragments vastly outnumbers miRNAs. This gives us a great opportunity to study tRNA expression using miRNA sequencing data through their anticodon sequences.

## Methods

### Ethics statement

This study was conducted in accordance with the Declaration of Helsinki with the approval of the Institutional Review Boards at Roswell Park Cancer Institute and Vanderbilt University Medical Center.

### Consent statement

Written informed consent for the patient-derived specimens was obtained prior to the acquisition in all cases. The consents were approved by the Institutional Review Boards at Roswell Park Cancer Institute and Vanderbilt University Medical Center.

### Specimen description

Bone marrow aspirate specimens (*N* = 69) (see Additional file 1: Table S1) were obtained from the Roswell Park Cancer Institute with appropriate approval by the Institutional Review Boards at Roswell Park and Vanderbilt University Medical Center. Twenty-two of the 69 bone marrow samples were from control patients (staging marrows that were negative for hematolymphoid malignancy). The other 47 samples were from MDS patients. Out of 47 MDS samples, 24 were pre-treatment specimens, and 23 were obtained after treatment with 5-azacytidine or decitabine, both DNMTIs. For each of the pre- and post-treatment paired samples, a response score (range: 1–6, where 1 represents complete remission, 2 complete marrow remission, 3 partial remission, 4 hematologic improvement, 5 stable disease, and 6 progressive disease) was obtained based on clinical and pathological criteria to indicate the effectiveness of treatment (Additional file 1: Table S1) [56]. Bone marrow mononuclear cells (BM-MNCs) were isolated from fresh, unsorted bone marrow aspirate specimens (*N* = 69) by the Ficoll method using Cellgro Lymphocyte Separation Medium (Corning, Manassas, VA). After performing a cell count, the cells were re-pelleted from Dulbecco’s phosphate buffered saline solution and resuspended in Gibco Recovery Freezing medium (Invitrogen, Grand Island, NY), and then frozen gradually, prior to storage in liquid nitrogen. Cells were frozen at a density of 10 to 20 million cells per mL.

### RNA isolation and RNA sequencing

Total RNA (totRNA) was isolated using a mirVana miRNA isolation kit (Life Technologies, Grand Island, NY) per manufacturer’s instructions. Sequencing library construction was performed on the totRNA from all 69 samples, each obtained from a single bone marrow aspirate specimen. RNAs were isolated by the mirVana RNA isolation kit, which was found to perform better than the TRIzol miRNA isolation kit [13]. Libraries were prepared using the TruSeq Small RNA sample preparation kit (Illumina, San Diego, CA). The small RNA protocol specifically ligates RNA adapters to mature miRNAs that have a 5’-phosphate and 3’-hydroxyl group resulting from enzymatic cleavage by RNA processing enzymes like Dicer. In the first step, RNA adapters were ligated onto each end of the RNA molecules, and a reverse transcription reaction was used to create single-stranded cDNA. This cDNA was then PCR amplified with a universal primer and a second primer containing one of 48 uniquely indexed tags to allow multiplexing. Size selection of the cDNA constructs was performed using a 3 % gel cassette on the Pippin Prep (Sage Sciences, Beverly, MA) to reduce the library to mature miRNAs and other regulatory RNAs in the 20–30 bp size range and to remove adapter-adapter products. The resulting cDNA libraries then underwent a quality check on the Bioanalyzer HS DNA assay (Agilent, Santa Clara, CA) to confirm the final library size and on the Agilent Mx3005P quantitative PCR machine using the KAPA library quantification kit (Illumina, San Diego, CA) to determine concentration. A 2 nM stock was created, and samples were pooled by molarity for multiplexing. From the pool, 10 pM was loaded into each well for the flow cell on the Illumina cBot for cluster generation. The flow cell was then loaded onto the Illumina HiSeq 2500 utilizing v3 chemistry and HTA 1.8. The raw sequencing reads in BCL format were processed through CASAVA-1.8.2 for FASTQ conversion and demultiplexing. The RTA chastity filter was used, and only the PF (pass filter) reads were retained for further analysis.

### Sequencing data analysis

We implemented a custom analysis pipeline for small RNA sequencing data processing. We used Cutadapt [57] to trim 3’ adapters for raw reads. Quality control on raw data was performed using QC3 [58]. All reads with length less than 12 were discarded. The adaptor-trimmed reads were formatted into a non-redundant FASTQ file where the read sequence and copy number was recorded for each unique tag. The usable unique reads were mapped to the whole genome using Bowtie1 [59] with only one mismatch allowed. The latest Sanger microRNA database, miRBase20 [60], was used to quantify microRNA isomiRs by reads mapped with position 0, +1, and +2 from the 3’ terminal of the miRBase locus. The tRNA database was prepared by combining the latest UCSC tRNA database with the tRNA loci of mitochondria from the ensembl database [61]. The reads mapped with tRNA loci were used not only for tRNA quantification, but also for tRNA mapping position coverage analysis. The reads mapped to miRNAs, tRNAs, and other small RNAs (including lincRNAs, snoRNAs, snRNAs, rRNAs, and misc_RNAs in the ensembl database) were used for response category analysis. In addition, single nucleotide variations (SNVs) can also be detected through RNA-seq methods [14, 62–64]. We identified SNVs in the tRNA reads using VarScan 2 [65] and filtered the SNVs based on the VarScan 2 recommended filters. Inconsistent SNVs between paired samples were removed from analysis. Only SNVs with zero appearance in control samples were included in the analysis.

### Response analysis

A linear regression model was fit using response scores as outcomes and tRNA expression as predictors. Differential expression between MDS biopsy samples and control samples were conducted using MultiRankSeq [66], which is based on the combination of results from three distinct RNA-seq analysis packages: DESeq [67], edgeR [68], and baySeq [69]. A tRNA was considered significantly differentially expressed if the adjusted p-values for all three analysis packages were less than 0.05. The elastic net method [70] was used to select a panel of tRNAs that together explain a large proportion of the variation in patient response. Elastic net regularization [71] is a technique that conbines the L1 and L2 penalties of the lasso and ridge regression methods. The elastic net method provides variable selection to produce parsimonious and interpretable models in the *p* > > *N* case without being severely limited by the sample size. This method greatly improves performance in the case of highly correlated predictor variables (as we expect to have in clinical data), through the identification of groups of phenotypes with significantly high correlation that contribute the most to the variation in the data. Unsupervised hierarchical cluster analysis was conducted using HeatMap3 [72]. Functional analysis of tRNAs/tDRs was carried out manually by searching online due to the lack of a functional database for tRNA. In addition, a Fisher’s exact test was used to identify SNVs associated with MDS.

## Results

**Fig. 1:**
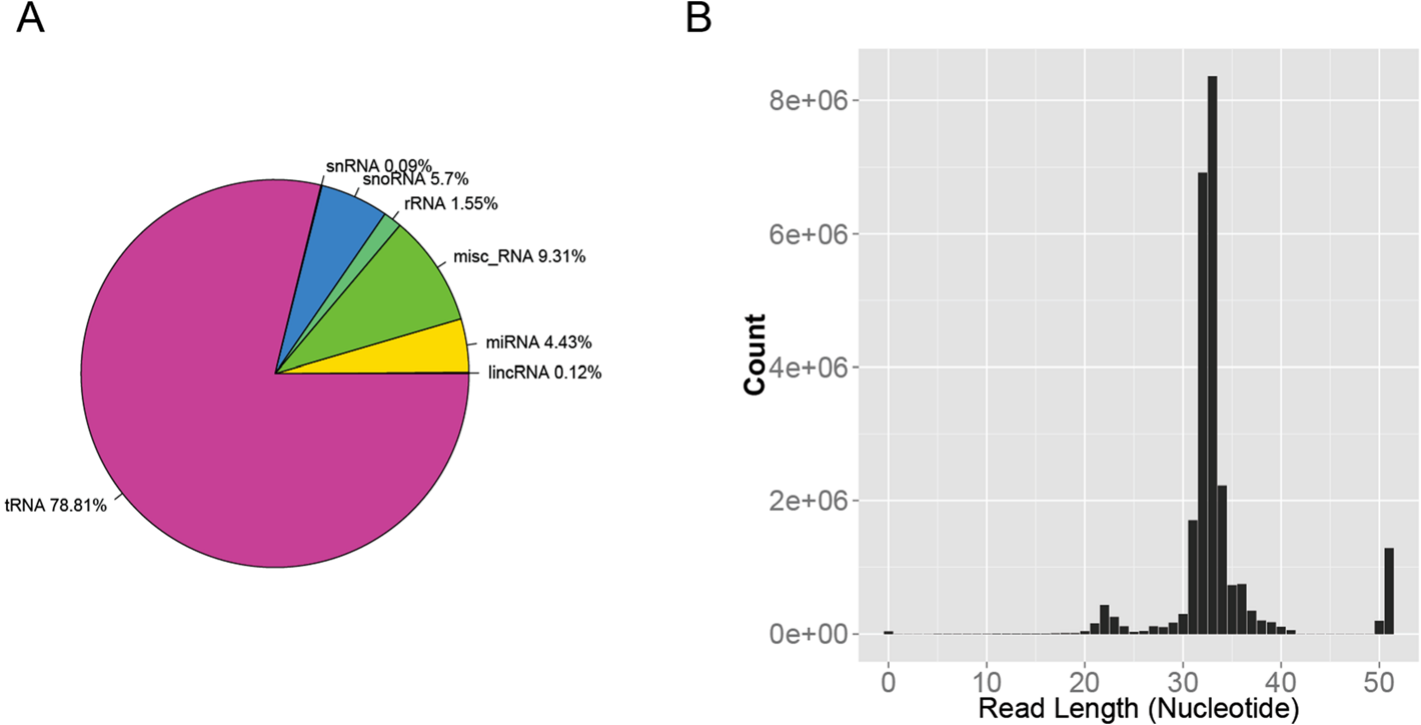
Read count and alignment distribution example taken from one sample. The figures were produced using all read counts per category, not just unique reads per category. The other samples in this study follow a similar pattern. **a**. Read count distribution after trimming adaptors. The smaller peak at 22 base pairs indicates the abundance of miRNA and the larger peak at 33 base pairs indicates the abundance primarily of tRNA. **b**. The reads alignment distribution by RNA type. The majority of the reads aligned to tRNA instead of miRNA

We found that for the reads obtained using a standard miRNA-seq protocol, the number of reads aligned to tDRs (78.81 % of reads) vastly outnumbered those aligning to miRNAs (4.43 % of reads) (Fig. 1a). The read length distribution, after trimming the adaptor with Cutadapt [57], shows a strong modal peak at 33 nucleotides, with the expected 22 nucleotide mean length of miRNAs (Fig. 1b) forming only a small secondary peak in the distribution. The peak at 33 bases indicates the abundance of tRNA species that have been cleaved in the anticodon loop from their full length of 73–94 nucleotides [10, 19]. The complete sequenced position of each tRNA is provided in Additional file 2: Figure S1. Numerous small RNA mapping statistics can be located in (Additional file 1: Table S2) (Fig. 1b).

**Fig. 2:**
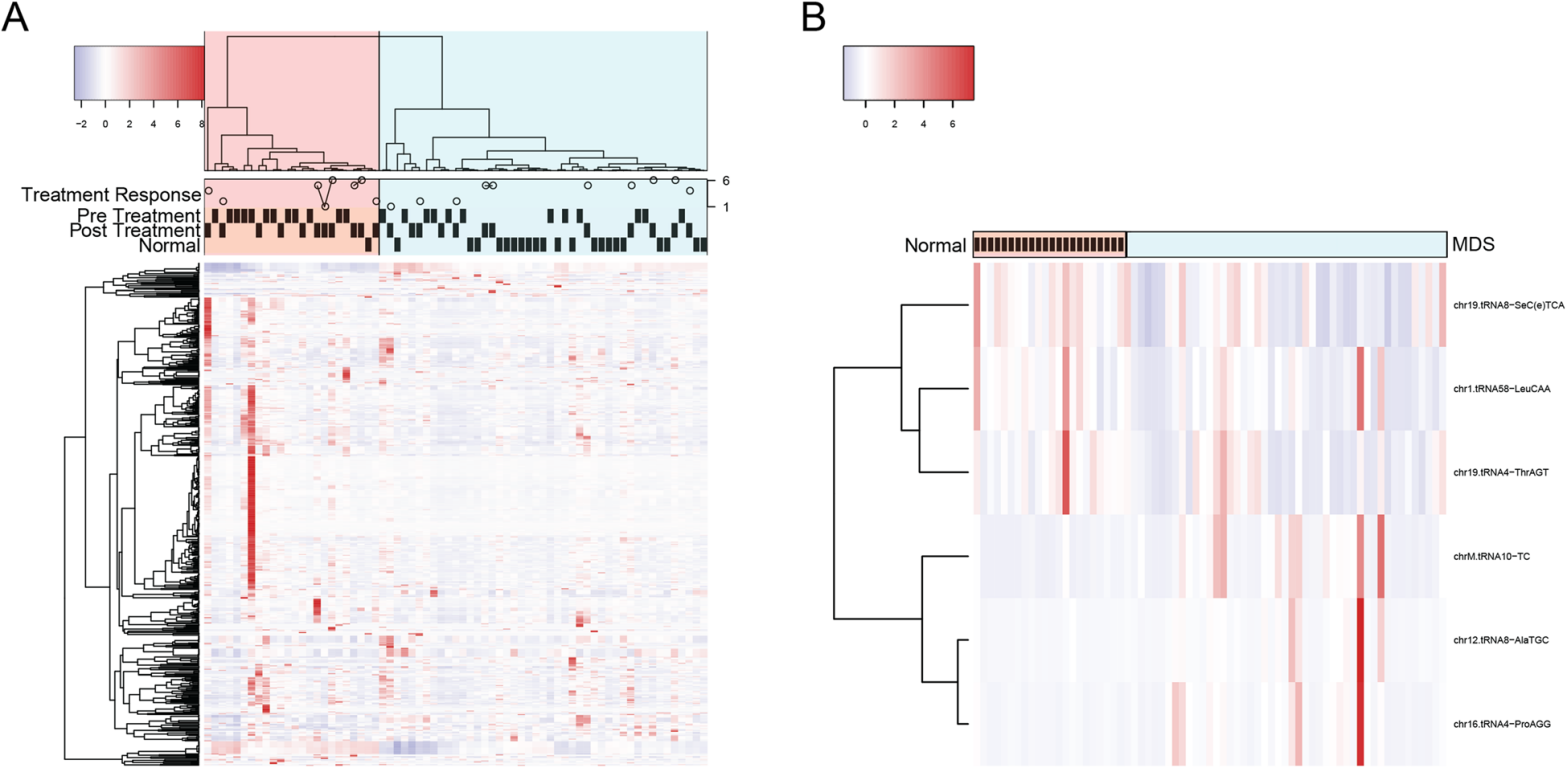
**a**. Cluster analysis and heatmap using tRNA expression of all samples. Three phenotype bars are drawn below the dendrogram: pre-treatment, post-treatment and normal controls. Two clusters are visible (light green and light red). These two clusters do not separate pre- and post-treatment, but distinguish MDS and normal samples reasonably well. **b**. The six differentially expressed tRNA between disease and normal

Using MultiRankSeq, we identified six tDRs that are significantly differentially expressed between MDS samples and control samples (Table 1, Fig. 2). Three tDRs demonstrated increased expression in MDS (chrM.tRNA10.TC, chr12.tRNA8.AlaTGC, and chr16.tRNA4.ProAGG) while three were decreased (chr1.tRNA58-LeuCAA, chr19.tRNA8-SeC(e)TCA (SeC(e)TCA), and chr19.tRNA4-ThrAGT). The complete results can be viewed in Additional file 1: Table S3. Unsupervised cluster analysis showed that tRNA profiling was able to distinguish between MDS and control samples (*χ*^2^*p* < 0.0001). No tDRs were significantly differentially expressed between pre- and post-treatment samples (Additional file 1: Table S4, Fig. 2).

**Table 1:** Differentially expressed tRNA derivatives (MDS versus control samples)

tRNA	log2FC^a^ DESeq2	pAdj^b^ DESeq2	log2FC edgeR	pAdj edgeR	pAdj baySeq^c^
chrM.tRNA10-TC	1.2840	0.0006	2.5347	0.0005	0.0011
chr12.tRNA8-AlaTGC	0.8518	0.0034	1.9498	0.0005	0.0378
chr16.tRNA4-ProAGG	0.8072	0.0274	1.6531	0.0043	0.0089
chr1.tRNA58-LeuCAA	−1.0706	0.0000	−0.7228	0.0406	0.0000
chr19.tRNA8-SeC(e)TCA	−0.6461	0.0103	−0.5944	0.0289	0.0126
chr19.tRNA4-ThrAGT	−0.8098	0.0067	−0.7906	0.0403	0.0489

^a^log2FC = log 2 fold change

^b^pAdj = adjusted p -value

^c^DESeq2, edgeR, and baySeq are the three RNAseq differential expression analysis packages used in this analysis. BaySeq does not generate fold change, thus no fold change from baySeq can be reported

Using the elastic net with ten-fold cross-validation (CV) for selecting the model with the smallest CV error, we identified a panel of four tRNA fragments (chr6.tRNA157.ValCAC, chr11.tRNA17.ValTAC, chrM.tRNA12.TS1, and chrX.tRNA4.ValTAC) whose combined expression in the pre-treatment samples together are predictive of the likelihood of response. By fitting a multivariable linear regression model using expression values of these four tDRs, two out of the four tRNA species showed significant associations with response (chr6.tRNA157.ValCAC *p* = 0.03564 and chrM.tRNA12.TS1 *p* = 0.01362) adjusted for other variables in the model. Overall, the model explained roughly 67 % of the variation in treatment response (R^2^ = 0.6735) (Table 2). It should be noted that the first 33 nucleotides of chr11.tRNA17.ValTAC and chrX.tRNA4.ValTAC show perfect sequence homology, so these different tRNAs cannot be distinguished by these methods. Therefore, it is possible that one or the other species are predictive of response rather than both.

**Table 2:** Linear regression results on tRNAs and treatment association

	tRNA	Effect^a^		p value
Treatment Response R-squared = 0.67	chr6.tRNA157.ValCAC	0.0149		0.0356
	chr11.tRNA17.ValTAC	0.1415		0.8343
	chrM.tRNA12.TS1	−0.4023		0.0136
	chrX.tRNA4.ValTAC	−0.0058		0.9931
Post vs Pre R-squared = 0.40	chr1.tRNA35.GlyGCC	−12.9856		0.3134
	chr19.tRNA9.PseudoTTT	0.0038		0.0507
	chr21.tRNA2.GlyGCC	12.9798		0.3136

Notice that not all tRNA are significant in this table; however, acting together, they explain the greatest amount of variation in treatment

^a^Effect is explained as per 1 unit increase in tRNA expression; the treatment changes the effect amount of unit

Using the change of tDR expression between pre- and post-treatment (computed as percentage of change), we conducted the same statistical analysis. Three tDRs (chr1.tRNA35.GlyGCC, chr21.tRNA2.GlyGCC, and chr19.tRNA9.PseudoTTT) were identified as predictors for treatment response. Multivariate linear regression was fit using treatment response as the outcome and tDR expression changes in the three tDRs as predictors. PseudoTTT trended towards an association with treatment response (*p* = 0.0507). Roughly 40 % of the variation in patient responses can be explained using the difference of expression between pre- and post-treatment of these three tDRs (R-squared = 0.4056) (Table 2). It should be noted that the first 33 nucleotides of chr1.tRNA35.GlyGCC and chr21.tRNA2. GlyGCC are also homologous, leading to alignment ambiguity.

Functional correlation of tRNA expression is challenging due to the dearth of available public resources focused on tRNAs. We focused on mitochondrial tRNAs (mt-tRNAs) as a limited subset of tRNA species to study, in particular those mt-tRNAs that have a demonstrated disease association in the literature (see references listed in Table 3). In Table 3, we show the false discovery rate (FDR) p-values for the ability of these tDRs from differential expression analysis methods to distinguish between MDS and control (Table 3, columns 3–5) and their association with treatment response (Table 3, columns 6–8) for all 22 mt- tDRs. Thirteen of the 22 mt-tDRs had significantly adjusted p-values from at least two of the three methods from MultiRankSeq in the discrimination of MDS from controls. MT-TS1 was the only tRNA from this subset that had significant association with treatment response.

**Table 3:** Test statistics of previously identified mitochondria tRNAs with disease associations

		Differential expression of tRNA MDS vs control			Linear regression association of tRNA with response		
tRNA	Association	pAdj (DESeq2)	pAdj (edgeR)	pAdj (baySeq)	Effect	Std err	p value
MT-TF	MELAS, MT-TF-related, MERRF syndrome [88]	0.0103	0.0015	0.4869	−1.3378	3.5754	0.7129
MT-TV	Leigh syndrome, NARP, mitochondrial disorder [89]	0.0103	0.0020	0.6661	−0.1755	0.2952	0.5602
MT-TL1	myelodysplastic syndrome [39]	0.0299	0.0030	0.4143	0.7728	1.1559	0.5127
MT-TI	hypertrophic cardiomyopathy [90]	0.0431	0.0044	0.7070	−1.3977	2.8147	0.6258
MT-TQ	MELAS [91]	0.4745	0.0687	0.9095	−0.1673	0.2261	0.4693
MT-TM	mitochondria disorder, hypertension [92]	NA	0.0354	0.6065	−0.6015	0.2818	0.0476
MT-TW	neonatal onset mito disease [93]	0.0271	0.0040	0.4824	−18.3065	27.0968	0.5084
MT-TA	hearing loss [94]	0.0062	0.0015	0.2336	−33.3062	19.0142	0.0979
MT-TN	Ophthalmoplegia [95]	0.0134	0.0024	0.5159	−6.6839	3.0347	0.0417
MT-TC	hearing loss [96]	0.0006	0.0005	0.0011	−0.7931	1.1525	0.5006
MT-TY	mitochondrial cytopathy [97]	0.5648	0.0642	0.9019	0.0994	3.1135	0.9749
MT-TS1	hearing loss [98]	0.0017	0.0009	0.1043	−54.1283	17.2749	0.0061
MT-TD	mitochondrial myopathy [99]	NA	0.1135	0.8845	−14.0023	9.2086	0.1467
MT-TK	MELAS [100]	0.6851	0.1933	0.9098	−0.4508	0.5028	0.3825
MT-TG	hypertrophic cardiomyopathy [101]	0.0775	0.0067	0.7119	0.4488	4.0525	0.9131
MT-TR	mitochondria myopathy [102]	0.4745	0.1346	0.8194	−19.9639	10.4921	0.0742
MT-TH	MELAS [103]	0.1634	0.0251	0.8843	−0.4952	0.4202	0.2549
MT-TS2	mitochondrial myopathy [104]	0.0431	0.0043	0.8107	−0.0547	0.2504	0.8295
MT-TL2	ophthalmoplegia [105]	0.3483	0.0534	0.9062	−1.1874	1.2812	0.3670
MT-TE	myopathy [106]	0.0271	0.0045	0.5381	−0.3868	0.2411	0.1271
MT-TT	multiple sclerosis [107]	0.3483	0.0466	0.8629	−9.0631	11.8617	0.4553
MT-TP	mitochondrial catopathy [108]	0.0034	0.0007	0.4374	−4.2378	3.7122	0.2694

Through SNV analysis, we identified 14 SNVs that have potential associations with MDS (Table 4). These 14 SNVs were found in at least 20 % of MDS samples and were not observed in any of the control samples. Only one out of the 14 SNVs has been previously identified (rs192094984) by the 1000 Genomes Project. Some of the SNVs were identified in the same tRNA with the same anticodon but were located at different genomic positions. It is possible that they are the same SNV duplicated by alignment ambiguity.

**Table 4:** tRNA single nucleotide variant analysis

tRNA	Known SNP	CHR	POS	REF	ALT	Control with SNV	Control without SNV	MDS with SNV	MDS without SNV	Fisher p value
chr13.tRNA1-PheGAA	No	13	95201919	T	A	0	11	4	3	0.01
chr12.tRNA11-PheGAA	No	12	125412404	T	A	0	16	4	6	0.01
chr6.tRNA44-SerAGA	No	6	27446616	G	A	0	21	5	13	0.01
chr6.tRNA46-SerAGA	No	6	27463618	G	A	0	21	5	13	0.01
chr6.tRNA47-SerAGA	No	6	27470843	G	A	0	21	5	13	0.01
chr6.tRNA50-SerAGA	No	6	27500012	G	A	0	21	5	13	0.01
chr6.tRNA51-SerTGA	No	6	27513493	G	A	0	21	5	13	0.01
chr6.tRNA5-SerAGA	No	6	26327842	G	A	0	21	5	14	0.02
chr6.tRNA148-SerTGA	No	6	27473663	C	T	0	21	5	14	0.02
chr6.tRNA147-SerAGA	No	6	27509610	C	T	0	21	5	14	0.02
chr17.tRNA35-SerAGA	No	17	8129984	C	T	0	21	5	14	0.02
chr6.tRNA172-SerTGA	No	6	26312880	C	T	0	21	5	15	0.02
chr7.tRNA21-CysGCA	rs192094984	7	149112285	G	A	0	16	3	7	0.05
chr8.tRNA11-SerAGA	No	8	96281941	C	T	0	21	5	17	0.05

## Discussion

In this study, using miRNA sequencing data generated from 69 BM-MNC samples and a novel bioinformatics approach, we demonstrated that tDRs are a major byproduct of miRNA sequencing, exceeding the abundance of miRNAs detected by this methodology by nearly 18-fold. This finding demonstrates the difficulties inherent in studying low-abundance RNA species by expression analysis and emphasizes the importance of adequate filtration.

Due to the potential information embodied in the tDR dataset and a lack of published data on the topic, we chose to study the expression of tDRs in MDS. We demonstrated that six tDRs are differentially found in MDS and control samples, and that a pattern of tRNA species could be used to differentiate MDS and control samples using unsupervised hierarchical clustering. Most significantly, the expression of tDRs in pre-treatment samples was found to predict response to treatment with DNMTIs. These tDRs were found to explain 67 % of the variation in treatment response. The expression of these entities could be used in the clinical setting to select patients likely to respond to DNMTIs. Since only a minority of patients achieve a clinically meaningful response of hematologic improvement, partial response, or complete response to DNMTI therapy, and since it typically takes 3–6 cycles before a response is evident [48], pre-treatment identification of patients likely not to benefit from DNMTI therapy would enable earlier decisions about stem cell transplantation or investigational therapies in those patients. Using changes in tDR expression between pre- and post-treatment samples in model fitting would explain less variation in the data (R^2^ = 40 %); however, since pre-treatment tRNA expression is a more clinically useful analysis, the better prediction value is fortuitous. Unfortunately, due to the small size of this study, internal validation methods have limited utility. The optimal method to demonstrate the sensitivity and specificity of these biomarkers is to utilize an independent dataset. This will be the focus of future efforts.

Due to the recent developments in the study of mt-tRNAs and their association with various human diseases [60], the intriguing studies on mitochondrial aberrations in MDS [73], and the identification of chrM.tRNA12.TS1 and chrM.tRNA10.TC in our initial analyses, we examined the expression of mt-tRNA-derived species in MDS. We identified 22 mt-tDRs which individually are differentially expressed in MDS compared to control samples, as well as in responders and non-responders to DNMTI therapy. Again, these may be valuable diagnostic and prognostic markers in MDS; however, the correlation between this functional subset tDRs and the mitochondrial aberrations seen in MDS is unclear.

Finally, we found increased SNVs within the tRNA sequences in the MDS samples as compared to the controls. While this may simply point to underlying genomic instability of myeloid neoplasms, studies in acute myeloid leukemia have not generally found increased absolute numbers of mutations [74], and many mutations are found as a result of aging alone in individuals without any significant hematolymphoid malignancy [75–77]. Therefore, there may be real functional consequences of these SNVs which may affect the functions of these tRNA-derived species. It should be noted that RNA-seq is not a well-accepted method for the identification of SNVs due to the high error rate of the reverse transcription step and the RNA editing process [78, 79]. In addition, the presence of numerous modified nucleotides in tRNAs (e.g. methylation) can result in base errors during the reverse transcription step of RNA-seq [22]. However, these SNVs were not identified to an appreciable level in the control samples, which were processed identically in a blinded fashion, suggesting that the significance of this finding is not an artifact.

Study of tRNA fragments using miRNA sequencing data is not without limitation. As we described previously, tRNAs are typically 73–94 nucleotides in length, composed of three hairpin-turns in a cloverleaf two-dimensional structure. The most 5’ of these loops is the D loop. Within this loop there is a site that has been shown to be sensitive to cleavage either by Dicer or by simple incubation with MgCl_2_ to afford a 19 nucleotide product from the 5’ end [10]. However, figures in this same publication also identify a cleavage product of greater than 30 nucleotides [10] that is too small to correspond to the residual 3’ fragment. As we have shown, 33 nucleotide tRNA derivatives were identified by our sequencing efforts, resulting from cleavage within the second hairpin loop containing the anticodon. Our size selection step would have excluded most of the 19 nucleotide product. In addition, cleavage in the anti-codon loop occurs physiologically as a result of cleavage by angiogenin [18] and may be targeted by other nucleases as well [19]. Lastly, due to the poor reverse transcription of full length parent tRNAs due to nucleotide modifications, apparent tDRs may result from incomplete reverse transcription [23]. Thus, for various biological and technical reasons, it is possible that under the conditions of this study, the 33 base derivative may be the predominant 5’ tDR product.

The lack of knowledge of the entire sequence of the tRNA creates ambiguous tRNA annotation. Many of the tRNA isotypes have not only the same or similar anticodons, but also highly homologous sequences 5’ to the anticodon. This creates challenges in the identification of specific species that may be diagnostically or prognostically useful. As mentioned in the results, several of the prognostic tRNA fragments are homologous. Therefore, although the degenerate species are individually identified, the prognostic power may lie in only one of the entities. Also, in Table 4, a G > A SNV was identified in five SerAGA tRNAs. Since the first 33 base pairs of these five tRNAs are the same, the redundant alignment resulted in the identification of a G > A SNV in all five SerAGA tRNAs for the homologous position, when in fact it may have only been seen in as few as one of the five SerAGA isotypes. The actual nucleotide sequences of each of the tDRs specifically mentioned in this study are available for comparison in Additional file 1: Table S5. Furthermore, we used VarScan 2 to identify SNVs. Typically, at least 20 % of the reads need to support the alternative allele in order to be called an SNV by VarScan 2. So, although none of the control samples in Table 4 contained an SNV as determined by VarScan 2, there may have been low levels of reads that did not meet the 20 % threshold for identification of an SNV.

It is unclear if the expression of tDRs represents the true expression of the full-length tRNAs themselves, if they are physiologic by-products whose presence at different levels suggests differential processing and, therefore, half-lives of the tRNAs, or if they represent biologically active entities in themselves. Needless to say, this ambiguity complicates the further exploration of these results, requiring novel methods to unravel this unknown biology. Recently, new approaches to library preparation have emerged, including the use of demethylases and other strategies to remove modifications prior to reverse transcription. Moreover, the use of Group II intron reverse transcriptases, that bind more tightly than retroviral enzymes to the RNA template, has been demonstrated to overcome modification barriers to afford full-length tRNA cDNAs in RNA-seq libraries [80–83]. These methods may be useful in the future to distinguish the biological tDRs from those that result from technical artifact.

The exact mechanism by which tDRs may play a role in the diagnosis of MDS and prognostication of response to DNMTIs is unclear. There are multitudinous effects of global hypomethylation that results from DNMTI therapy. Some tDRs have been demonstrated to function independently as biologically active entities. Lee *et al*. have identified small tRNA-derived species, including tRF-1001, which promotes the G2/M transition [11]. However, the species identified by Lee *et al*. are typically 17–26 bases long, smaller than the 33 nucleotide reads identified in this study. Several reports suggest that 30–35 nucleotide long tRNA-derived fragments may play a role in the biogenesis of other small RNAs, and these have been found in wide ranges of species, including humans. Yamasaki *et al*. suggest that these fragments may mediate stress-related translational repression [84]. However, these explanations would have unclear biologic importance in the differential expression of specific tRNA-derived sequences. Alterations in the activity of DICER, and hence of miRNAs, are a common feature in cancers [85–87]. tRNAs have been proposed as alternative DICER1 substrates [39]. Whether certain tRNAs are selectively targeted by DICER1 in cancer in general, and MDS in particular, remains to be seen, but it provides an intriguing hypothesis for the differentially expressed tDRs identified in this study in association with both the diagnosis and prognosis of MDS.

## Conclusions

This study suggests that tRNA-derived fragment sequencing can provide an additional source of data that potentially provides clinically useful applications in diagnosis and prognosis of disease. In addition, this study raises intriguing questions about the biology of these tRNA derivatives in MDS and other cancers.

## Additional files

Additional file 1: Table S1. Sample description. **Table S2.** Phenotype and alignment results for all samples. **Table S3.** Differential expression analysis between MDS and control samples. **Table S4.** Differential expression analysis between pre and post treatment. **Table S5.** Nucleotide sequences of the tRNAs described in this paper. (ODS 152 kb) Additional file 2: Figure S1. Using a single sample as an example, this figure shows the alignment position of each tRNA. The majority of the alignment lies between residues 1–33 from the 5’ end. The size of each dot indicates the abundance of reads aligned to this position. (PNG 21 kb)

## Abbreviations

miRNA: microRNA
miRNA-seq: microRNA sequencing
MDS: Myelodysplastic syndromes
tRNA: Transfer RNA
tRHs: Transfer RNA halves
tDRs: Transfer RNA-derived small RNAs
nt: Nucleotide
AML: Acute myeloid leukemia
DNMTIs: DNA methyltransferase inhibitors
BM-MNCs: Bone marrow mononuclear cells
totRNA: Total RNA
SNV: Single nucleotide variant
CV: Cross-validation
mt-tRNAs: Mitochondrial transfer RNAs
FDR: False discovery rate
