# Supplementary figures and images for: Transfer RNA detection by small RNA deep sequencing and disease association with myelodysplastic syndromes
Source: BMC Genomics. 2015 Sep 24;16:727. doi: 10.1186/s12864-015-1929-y (PMC4581457; doi:10.1186/s12864-015-1929-y)

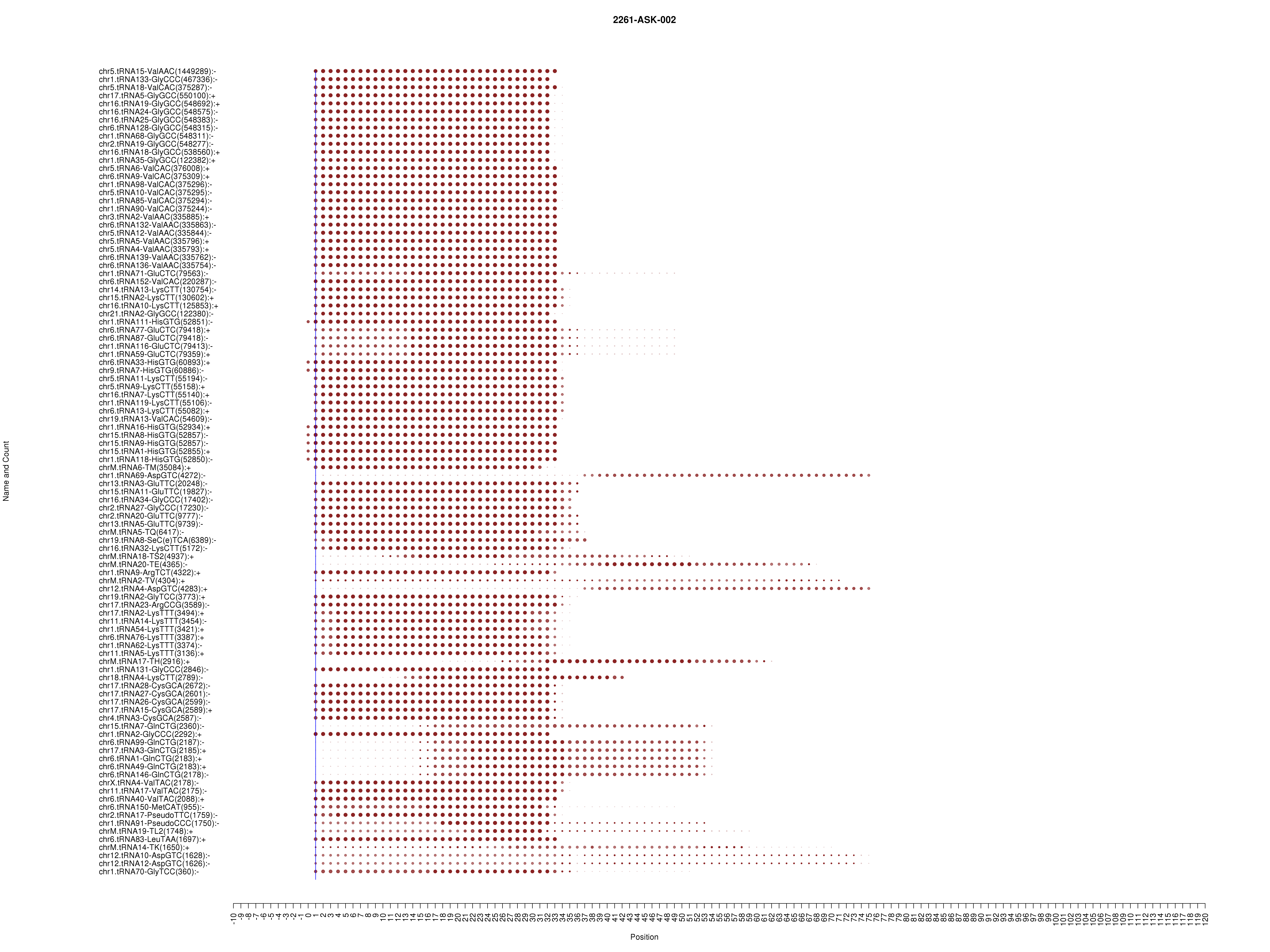

Supplement: Additional file 2: Figure S1. — Using a single sample as an example, this figure shows the alignment position of each tRNA. The majority of the alignment lies between residues 1–33 from the 5’ end. The size of each dot indicates the abundance of reads aligned to this position. (PNG 21 kb) [file 12864_2015_1929_MOESM2_ESM.png]
